# Wellness for Older Adults Living With Serious Mental Illness: A Proposed Practical Framework

**DOI:** 10.1093/geroni/igaa057.1525

**Published:** 2020-12-16

**Authors:** Michelle Zechner, Matthew Fullen, Nora Barrett, Margaret Swarbrick, Carlos Pratt, Stephanie Santos

**Affiliations:** 1 Rutgers, RBHS, Piscataway, New Jersey, United States; 2 Virginia Tech, Blacksburg, Virginia, United States; 3 Rutgers, Piscataway, New Jersey, United States; 4 Rutgers, PIscataway, New Jersey, United States; 5 Rutgers School of Health Related Professions, Piscataway, New Jersey, United States; 6 Rutgers University, Middlesex, New Jersey, United States

## Abstract

Multi-dimensional wellness is a holistic, person-centered clinical approach, that offers promise as a framework for improving the health and well-being of older adults. This approach can be especially helpful for older adults who are experiencing mental health conditions as it focuses on strengths, resilience and recovery comprehensively. Few clinical interventions have addressed wellness in older adults with mental health conditions (Zechner et al., 2019).The authors reviewed two existing wellness frameworks: 1) grounded in extensive practical interventions for people living with mental health conditions (Swarbrick, 1997; 2006; 2012; 2017), and 2) a theoretical framework informed by a systematic literature review (Fullen, 2019). The two frameworks were compared for usefulness in older adults living with mental health conditions using the concept analysis strategies of analyzing the overlap and contrasting characteristics of concepts with related ideas, and then proposing a merged operationalized definition and practical implementation suggestions based on the characteristics of the original dimension (Mackeroff et al., 2016). The two wellness models described similar dimensions, but also represented distinct areas. We propose a synthesized framework combining the models to best serve older persons living with mental health conditions. The framework holds eight distinct areas 1) Developmental 2) Cognitive/ Intellectual, 3) Physical 4) Emotional 5) Social/Relational, 6) Occupational/Vocational 7) Spiritual and 8) Contextual/Environmental/Financial. Inter-professional practical clinical strategies and policy suggestions will be presented for consideration. More work is underway to understand the salience of the proposed model for stakeholders and to empirically test how the proposed framework impacts outcomes.

